# Characteristic features of the SERA multigene family in the malaria parasite

**DOI:** 10.1186/s13071-020-04044-y

**Published:** 2020-04-06

**Authors:** Nobuko Arisue, Nirianne M. Q. Palacpac, Takahiro Tougan, Toshihiro Horii

**Affiliations:** 1 Research Center for Infectious Disease Control, Research Institute for Microbial Diseases, Osaka University, Suita, Osaka 565-0871 Japan; 2 Department of Malaria Vaccine Development, Research Institute for Microbial Diseases, Osaka University, Suita, Osaka 565-0871 Japan

**Keywords:** *Plasmodium*, SERA, Gene family, Function, Polymorphism

## Abstract

Serine repeat antigen (SERA) is conserved among species of the genus *Plasmodium*. *Sera* genes form a multigene family and are generally tandemly clustered on a single chromosome. Although all *Plasmodium* species encode multiple *sera* genes, the number varies between species. Among species, the members share similar sequences and gene organization. SERA possess a central papain-like cysteine protease domain, however, in some members, the active site cysteine residue is substituted with a serine. Recent studies implicate this gene family in a number of aspects in parasite biology and induction of protective immune response. This review summarizes the current understanding on this important gene family in several *Plasmodium* species. The *Plasmodium falciparum* (*Pf*)-*sera* family, for example, consists of nine gene members. Unlike other multigene families in *Plasmodium* species, *Pf-sera* genes do not exhibit antigenic variation. *Pf*-*sera5* nucleotide diversity is also low. Moreover, although *Pf-sera5* is highly transcribed during the blood stage of malaria infection, and a large amount is released into the host blood following schizont rupture, in malaria endemic countries the sero-positive rates for Pf-SERA5 are low, likely due to Pf-SERA5 binding of host proteins to avoid immune recognition. As an antigen, the N-terminal 47 kDa domain of Pf-SERA5 is a promising vaccine candidate currently undergoing clinical trials. Pf-SERA5 and Pf-SERA6, as well as *P. berghei* (Pb)-SERA3, and Pb-SERA5, have been investigated for their roles in parasite egress. Two *P. yoelii* SERA, which have a serine residue at the protease active center, are implicated in parasite virulence. Overall, these studies provide insight that during the evolution of the *Plasmodium* parasite, the *sera* gene family members have increased by gene duplication, and acquired various functions that enable the parasite to survive and successfully maintain infection in the host.
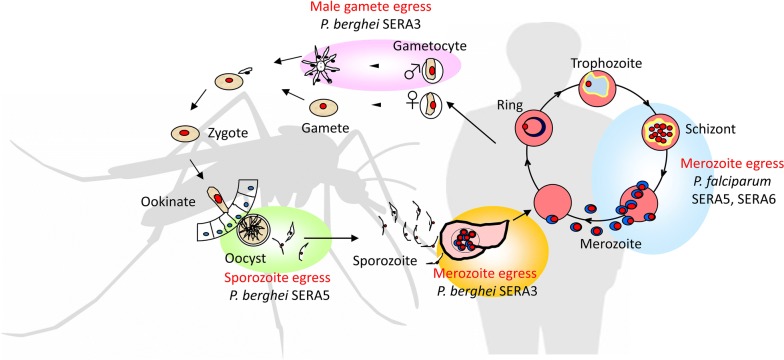

## Background

Malaria, transmitted by the bite of mosquitoes infected with the *Plasmodium* parasite, is a life-threatening infectious disease. In 2018, there were an estimated 228 million cases and 405,000 related deaths [1], most of which were children in the WHO African region. After an unprecedented decrease in the malaria burden following introduction of Coartem^®^, the decrease in malaria incidence and death has since reached a plateau, largely due to infrequent and unsustainable supply of current interventions. An effective malaria vaccine is urgently needed, but its development is extremely challenging due to several immune evasion mechanisms both in the mosquito vector and human host.

*Plasmodium falciparum* serine repeat antigen (Pf-SERA) is an asexual blood stage antigen, so named because of the stretch of serine residues found in its amino acid sequence [2]. *Pf-sera* was initially considered a single gene; however, the *P. falciparum* genome project revealed that *Pf-sera* resides in a multi-locus forming gene family. Eight genes, *Pf-sera1* to *Pf-sera8*, are clustered in tandem on chromosome 2 [3]. *Pf-sera9* was later found on chromosome 9 [4]. Notably, due to the N-terminal truncated form of Pf-SERA8, it was previously considered a pseudogene; however, further studies demonstrated that *Pf-sera8* was transcribed at the sporozoite stage [5]. The first identified Pf-SERA was *Pf-sera5*, which is the only family member containing a serine stretch. Its N-terminal 47 kDa domain (SE47) has been studied as a candidate vaccine antigen [6] and shows promise as (i) antibodies against this domain inhibit parasite growth *in vitro* [7–12]; and (ii) sero-epidemiological studies in malaria endemic areas have reported a negative correlation between parasitemia and anti-SE47 antibody titers [13, 14]. When originally designated as a multigene family, SERA were believed to be important for antigenic variation, which refers to the parasite’s ability to present a variety of antigenic molecules on the surface of infected red blood cells (RBC) to facilitate immune evasion [15]. However, the *Pf-sera* family, has been shown to not exhibit antigenic variation. Moreover, transcriptional analysis revealed that all *Pf-sera* genes, except for *Pf-sera8*, are transcribed nearly simultaneously [11].

All existing *Plasmodium* species have a *sera* multigene family; however, the number of genes varies between species [16, 17]. Gene duplication and gene loss have repeatedly occurred during the parasite’s evolution, allowing the parasite to undoubtedly develop new mechanisms for survival and maintenance of the delicate biological equilibrium of parasite and host. Following release of the *P. falciparum* draft genome, genome projects focused on several *Plasmodium* species have been accelerated resulting in characterization of the SERA multigene family structure in these species. In addition, functional studies have been performed at various points in the parasite life-cycle to better understand this multigene family. Herein, we review recent progress in these areas.

## Organization and evolutionary relationships of *sera* genes in 26 *Plasmodium* species

**Fig. 1:**
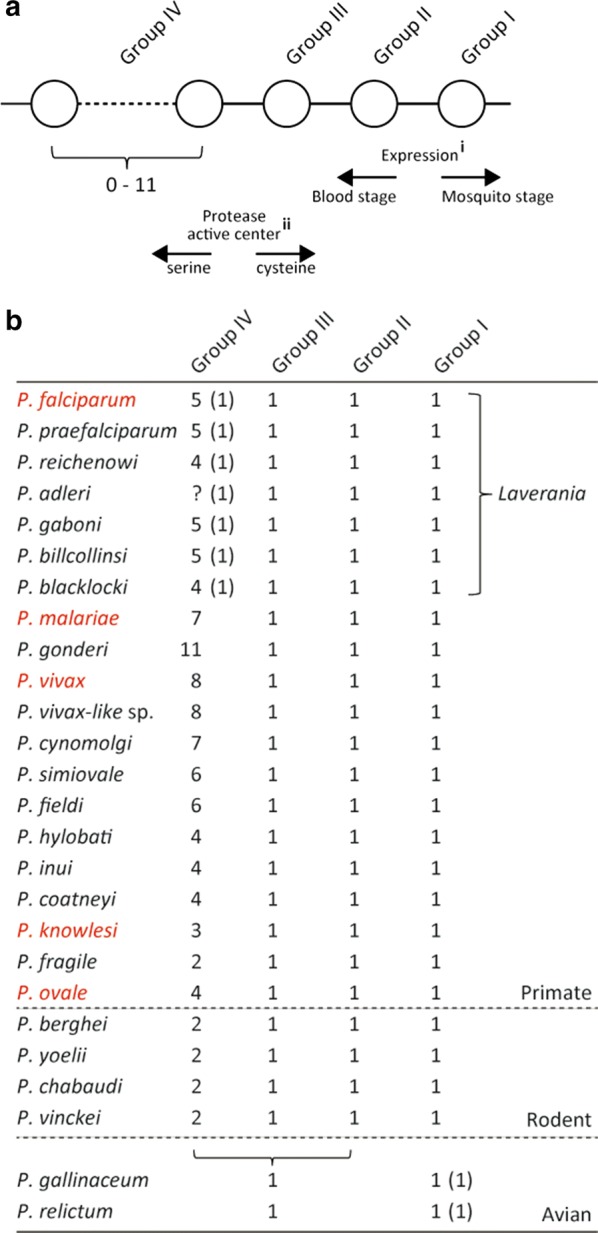
*Plasmodium sera* gene family. **a** The organization of *Plasmodium sera* genes and their characteristic gene and protein features. *Plasmodium sera* genes (denoted as circles) are clustered in tandem and are categorized into four groups based on genetic background. In terms of protein expression, i: Group I SERA is expressed in the mosquito stage and Group II to IV are expressed in the host blood stage. In terms of the protease motif, ii: SERA members containing cysteine residues in the protease active center belong to Groups I to III, while those with a substituted serine residue belong to Group IV. The number of Group IV SERA genes can vary from 1 to 11. **b** The number of *sera* genes identified in each group. For Group IV *sera* and two Group I *sera*, the number of genes not in tandem cluster is shown in parenthesis. *Plasmodium* species with humans as a natural host are shown in red. The number of Group IV *sera* genes in *P. adleri* is currently unresolved

The DNA sequence of *P. falciparum* chromosome 2 [3] provided the first insight into the *sera* multigene family. Eight genes, *Pf-sera1* to *Pf-sera8*, were clustered in tandem between two conserved genes: a hypothetical protein gene and the iron-sulfur assembly protein gene. Currently, the *Plasmodium* genome data, available in public databases, has more than 200 *sera* gene sequences from 26 *Plasmodium* species (Fig. 1, Additional file 1: Table S1). Each *Plasmodium* has multiple numbers of *sera* genes that are generally tandemly arranged on a chromosome in similar order as *Pf-sera1* to *Pf-sera8*. *Sera* genes found outside of the cluster, such as *Pf-sera9*, were found in avian malaria parasites and in *Laverania*, a subgenus of *Plasmodium*, which contain *P. falciparum* and related ape parasites. Among *Laverania* species, gene synteny was observed, with the location and arrangement of *sera* genes determined to be identical to that of *Pf-sera*, with the exception of *P. reichenowi* (chimpanzee parasite) and *P. blacklocki* (gorilla parasite), both of which lack the *Pf-sera3* orthologue [18]. Of note, the number of *sera* genes in *P. adleri* (another gorilla parasite) also remains uncertain due to ambiguous genome mapping of the *sera* gene region [18]. Furthermore, in the avian *P. relictum* (*Pr*), *Pr-sera1* and *Pr-sera2* are located in tandem on chromosome 4 while *Pr-sera3* is located on chromosome 9; meanwhile in the avian *P. gallinaceum* (*Pg*), the location of each gene has not yet been assigned to specific chromosomes. Nevertheless, *Pg-sera1* and *Pg-sera2* have been identified as being tandemly located on the same contig while *Pg-sera3* is located elsewhere.

**Fig. 2:**
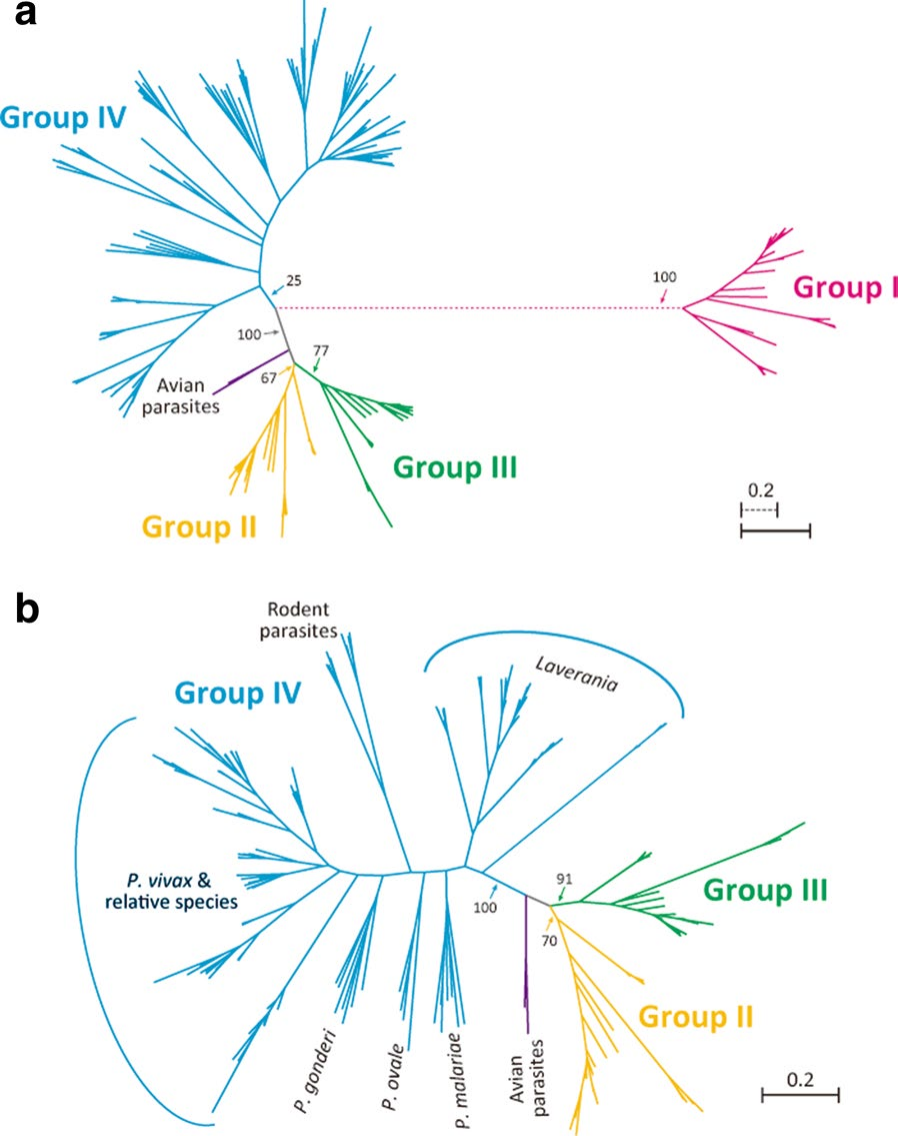
Phylogenetic tree of *sera* genes inferred by the maximum likelihood (ML) method. The sequences of *Plasmodium sera* genes were translated to amino acid sequences and aligned by using MAFFT version 7.409 [58]. Site selection for tree inference was done with the Gblocks option, with a more stringent selection criteria implemented in SeaView version 4.7 [59]. Trees were inferred under the JTT+Г+I model. MEGA X [60] was used for the analysis. Bootstrap proportions from the ML methods with 100 replications are shown. **a** 300 amino acid positions from 195 Group I to IV *sera* genes were used for tree inference. The tree with the highest log likelihood (−*li *= 28137.0426) is shown. A discrete Г distribution was used to model evolutionary rate differences among sites (4 categories, +Г, parameter = 1.1871). The rate variation model allowed for some sites to be evolutionarily invariable (+I, 9.2557% sites). **b** 424 amino acid positions from 173 Group II to IV *sera* genes were used for tree inference. The log likelihood of the tree is − 37969.9. A discrete Г distribution was used to model evolutionary rate differences among sites (4 categories, +Г, parameter = 1.2356). The rate variation model allowed for some sites to be evolutionarily invariable (+I, 5.0708% sites)

All of the SERA members contain a papain-like cysteine protease motif and can be classified into two major types based on the active site residue: a cysteine-type or serine-type SERA. Based on sequence similarity, *sera* genes can be categorized into four groups (Group I to IV) [16, 17] (Fig. 1). Group I to III contain cysteine-type SERA and Group IV contains a serine-type SERA. The *sera* gene IDs and their groupings are summarized in Additional file 1: Table S1. The maximum likelihood (ML) tree showed that each of the *sera* gene groups form monophyletic groups (Fig. 2a).

Transcription of Group I *sera* genes was detected primarily during the mosquito stage, while that of Group II to IV was observed during the blood stage in the vertebrate host [18]. Among 26 *Plasmodium* species, the number of *sera* genes in tandem cluster varied from 2 (in two avian parasites) to 14 (in *P. gonderi*, excluding a truncated *sera* gene [19]). Apart from avian parasites, the difference in the number of genes is always found in serine-type Group IV *sera* genes. The ML tree suggests that the generation of Group II and III *sera* genes would have occurred after the divergence of avian parasites, as *sera1* of the avian parasites is positioned at the branch leading to the common ancestors of Group II and III *sera* genes [16]. The ML tree also suggested that gene duplication(s) of the Group IV *sera* genes occurred independently in each lineage, generating multiple numbers of Group IV *sera* genes. The number of Group IV *sera* genes remarkably increased in some primate *Plasmodium* species (Figs. 1b, 2b). Like other multigene families, the *Plasmodium sera* multigene family was considered to be driven by birth-and-death evolution [17]. This model assumes that new genes are created by gene duplications, and that some duplicated genes can be maintained in the genome for a long time while other genes become deleted or non-functional through deleterious mutations [20]. Gene loss, gene truncation, and pseudogenization were observed in Group IV *sera* genes of primate parasites except for *P. malariae*, *P. ovale* and *Laveranian* species [17]. These processes may be associated with an evolutionary event that involves the acquisition of a new enzymatic pathway, expansion of host range and/or activation of new roles in host immune evasion.

## Functions of SERA

**Fig. 3:**
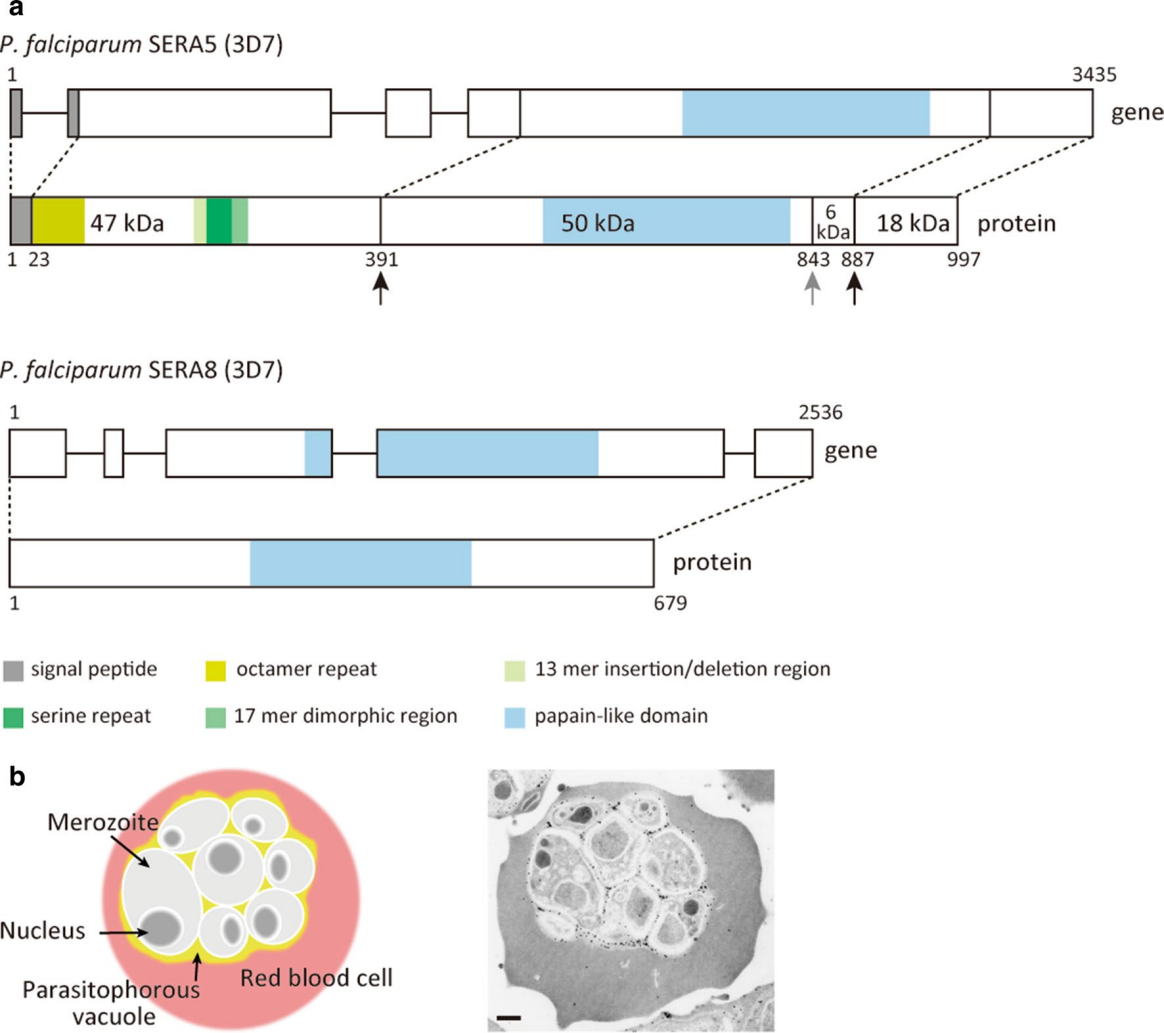
Structure and localization of SERA. **a** Schematic representation of the *Plasmodium falciparum* SERA5 and SERA8 genes and proteins; *P. falciparum* 3D7 strain was used as a reference for the illustration. The gene IDs in PlasmoDB [18] were PF3D7_0207600 and PF3D7_0207300, for Pf-SERA5 and Pf-SERA8, respectively. Amino acid positions for the Pf-SERA5 fragments are: 47 kDa corresponding to 23–390 aa; 50 kDa: 391–842 aa; 6 kDa: 843–886 aa; and 18 kDa: 887–997 aa. Black arrows indicate the PfSUB1 cleavage sites. The 50 kDa + 6 kDa fragment is further processed by an unknown protease (gray arrow). **b** Localization of Pf-SERA5 in the parasitophorous vacuole. Left image shows an infected red blood cell with schizont stage malaria parasites inside the parasitophorous vacuole. Right image, N-terminal antisera was used to localize Pf-SERA5 within a schizont parasitized red blood cell. Viewed under JEM-1230 transmission electron microscope (18,000× magnification). SERA5 conjugated gold particles were observed inside the parasitophorous vacuole. *Scale-bar*: **b**, 500 nm

The gene structure and primary protein structure of Pf-SERA5 (Group IV) and Pf-SERA8 (Group I) are shown in Fig. 3a. *Pf-sera5* is comprised of four exons and three introns. This exon/intron structure is common in most Group II to IV *sera* genes, with few exceptions. Alternatively, Group I of *Laverania* species, which includes *Pf-sera8*, is comprised of five exons and four introns, while all other *Plasmodium* Group I *sera* gene contain six exons and five introns [17]. In terms of protein structure, all *Plasmodium* SERAs contain a papain-like cysteine protease domain in the middle of the molecule; however, the canonical cysteine center is substituted with a serine in Group IV SERA. Group I SERA retains the conserved cysteine residue in the protease domain, and proteolytic activity was observed in rodent parasites [21]. The possibility of protease activity for Group IV SERA was dismissed by reverse genetics analysis of Pf-SERA5; as substituting the serine residue for alanine had no phenotypic consequence [22].

**Fig. 4:**
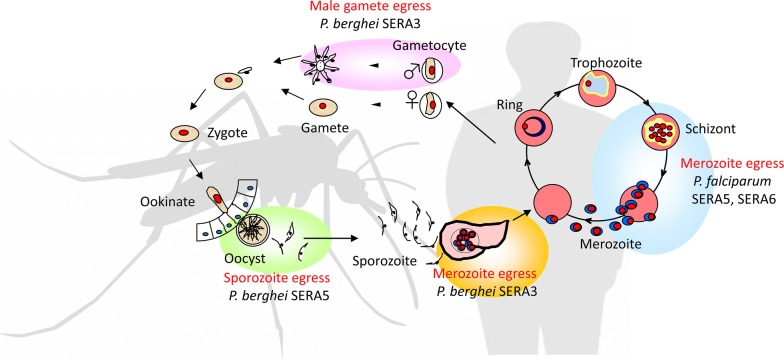
*Plasmodium* life-cycle and SERA function. The parasite life-cycle in the mosquito vector and human host. The colored circles indicate where SERA function is proposed by current studies

The role of SERA was primarily determined using *P. falciparum* and *P. berghei*. As shown in Fig. 4, SERA functions at several stages of the malaria parasite life-cycle, in both host and mosquito vectors. Knockout/disruption of nine *Pf-sera* genes suggested that *Pf-sera5* and *Pf-sera6* are indispensable in the asexual blood stage [23, 24]. In addition, conditional mutagenesis confirmed the necessity of *Pf-sera6* [25]. These data imply that Pf-SERA5 and Pf-SERA6 have crucial roles in malaria blood-stage infection.

### Asexual blood stage

Pf-SERA5 is a blood stage antigen expressed during late trophozoite and schizont stages as a 120 kDa precursor and secreted into the parasitophorous vacuole (PV) after removal of the signal peptide [26] (Fig. 3b). Pf-SERA5 is cleaved by an essential *P. falciparum* subtilisin-like serine protease 1 (PfSUB1) into 47 kDa, 56 kDa and 18 kDa fragments [27] (Fig. 3a). The 47 kDa fragment is linked to the 18 kDa fragment *via* a disulfide bond and localizes to the merozoite surface [28, 29]. The 56 kDa fragment containing the papain-like catalytic domain is further cleaved by an unknown protease to 50 kDa and 6 kDa fragments just before parasite egress [22, 27, 28]. Since the cleavage of the 56 kDa fragment is sensitive to cysteine protease inhibitors, for example E64, leupeptin and iodoacetoamide, the unknown protease is also believed to be a cysteine protease [28]. PfSUB1-mediated Pf-SERA5 processing is required for efficient egress from host erythrocytes [30].

Although protease activity of Pf-SERA5 has been refuted using native [31] and recombinant Pf-SERA5 [22], schizont rupture and merozoite release subsequently occur following Pf-SERA5 processing, and a block in the proteolytic processing of Pf-SERA5 is correlated to a block in erythrocyte rupture [27, 32–34]. Hence, Pf-SERA5 contributes an essential function that cannot be compensated by other members of the SERA family [22]. Furthermore, conditional disruption of Pf-SERA5 suggested its importance in regulating the kinetics and efficiency of parasite egress [35]. Pf-SERA5 also interacts with calcium dependent protein kinase 1 (PfCDPK1). Phosphorylation of Pf-SERA5 by PfCDPK1 boosts cytosolic Ca^2+^ levels, which serves as a trigger for merozoite egress [36].

Pf-SERA6, Group III, possesses the canonical cysteine residue in the active site cleft of the papain-like central domain, and is expressed simultaneously with Pf-SERA5 in the PV. Pf-SERA6 is also cleaved by PfSUB1, transforming Pf-SERA6 into an active cysteine protease [37]. Thomas et al. [38] showed that parasites lacking PfSUB1 fail to rupture the PV membrane (PVM). In contrast, Pf-SERA6-null parasites successfully ruptured the PVM yet failed to rupture RBC membrane to release the merozoites. Thus, Pf-SERA6 activated by PfSUB1 is required for the disassembly of the RBC cytoskeleton. Although, both Pf-SERA5 and Pf-SERA6 have been shown to be essential in parasite egress, the precise role and mechanism of Pf-SERA5 remains unclear.

The conserved nature of SUB1 across all *Plasmodium* species infers its importance to the parasite. In fact, the consensus sequence of the SUB1 cleavage site, (Val/Leu/Ile)-Xaa-(Gly/Ala)-Paa, where Xaa represent any amino acid residue and Paa denotes a non-polar residue, except for Leu [27], is well conserved in Group II to IV SERA [17]. SUB1 is also well conserved among *Plasmodium* species [39].

### Liver stage

*Plasmodium berghei* SUB1 (PbSUB1)-mediated processing activates *P. berghei* SERA3 (Pb-SERA3) [40]. Pb-SERA3, the orthologue of Pf-SERA6, is expressed in the late liver stage [40, 41] and is believed to function as a cysteine protease. Involvement in PVM rupture was suggested when disruption of the PVM did not occur in the presence of the cysteine protease inhibitor E64 [42]. Under E64-treatment, host hepatocytes and PVM were intact and no Pb-SERA3 secretion to the host cell cytoplasm was observed in *P. berghei*-infected cells [41]. The proteolytic processing is an essential process of PVM rupture in liver stage parasites [40]. In addition, it was suggested that Pb-SERA3 contributes to host cell death by activating other parasite or host proteins following PVM breakdown [41].

### Oocyst and sporozoite stages

The function of Group I SERA was analyzed using Pb-SERA5, the orthologue of Pf-SERA8. Pb-SERA5 is expressed in oocyst and sporozoite stages [16]. Midgut sporozoite egress from oocysts was prevented by disruption of Pb-SERA5 [21] (ECP1 in Aly et al. [21] is identical to Pb-SERA5). Pb-SERA5 does not have the conserved SUB1 cleavage site and the detailed underlying molecular mechanism remains unknown; however, the sequence of Group I is highly conserved among *Plasmodium* species. The timing of the expression of Group I SERA is similar between Pb-SERA5 and Pf-SERA8 [18]. Moreover, Pb-SERA5-knockout parasites affected circumsporozoite protein (CSP) processing, suggesting a possible involvement of Pb-SERA5 in CSP maturation [21]. Altogether, these data suggest that Group I SERA functions in sporozoite egress from oocysts.

### Gametocyte stages

It was also shown that PbSUB1 plays a critical role in male gamete egress [43]. During the process, Pb-SERA3 expressed in male gametocytes is cleaved by PbSUB1 when it is discharged into the PV. This proteolytic process mediates PVM rupture and parasite egress.

## Polymorphism of *sera* genes

**Fig. 5:**
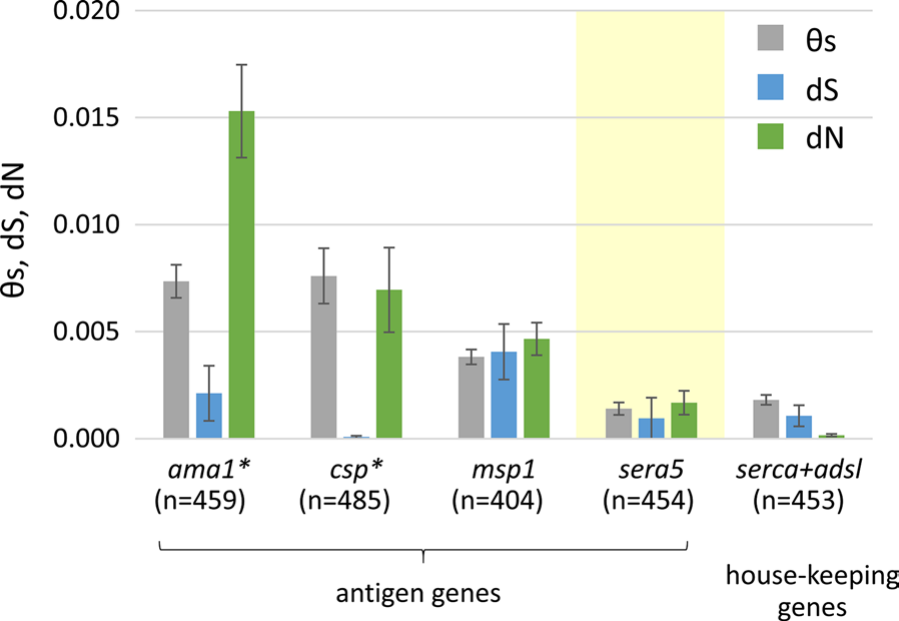
Polymorphism of *P. falciparum* antigen genes and housekeeping genes. The amount of nucleotide diversity expressed by the standardized number of polymorphic sites per site (θ_s_), the numbers of synonymous substitutions per synonymous site (dS), and non-synonymous substitutions per non-synonymous site (dN). Data were taken from Tanabe et al. [45, 47]. The asterisk denotes a significant difference between dS and dN (*P* < 0.01). *Abbreviations*: n, number of sequences analyzed; *ama1*, apical membrane protein 1; *csp*, circumsporozoite protein; *msp1*, merozoite surface protein 1; *sera5*, serine repeat antigen 5; *serca*, P-type Ca^2+^-ATPase; *adsl*, adenylosuccinate lyase

The malaria vaccine candidate antigen Pf-SERA5 has an octamer repeat and serine repeat at the N-terminal region (Fig. 3a). The number of both repeats vary among field isolates as well as laboratory parasite lines [44, 45]. In addition, there are 13-mer insertion/deletion and 17-mer dimorphic regions close to the serine stretch region [44, 45]. One epitope in the octamer region at amino acid positions 59–72 (PF3D7_0207600) [18] was shown to be the target of parasite growth inhibitory antibodies *in vitro* [8, 46] and was perfectly conserved in 445 worldwide isolates of *P. falciparum* [45]. Polymorphic sites in non-repeat regions of *Pf-sera5* (2562 bp) was limited to only 24 nucleotide sites. The ratio of dN (the number of non-synonymous substitutions per non-synonymous site), dS (the number of synonymous substitutions per synonymous site), and Tajima’s *D* test did not detect any strong signature for positive selection in the non-repeat regions of *Pf-sera5* [45]. The nucleotide diversity of non-repeat regions of *Pf-sera5* is comparable to the housekeeping genes of P-type Ca^2+^-ATPase (*serca*) and adenylosuccinate lyase (*adsl*) (Fig. 5). This is an advantage in the current efforts to develop an effective vaccine, since overcoming antigenic diversity remains a key challenge with most vaccine candidates tested in clinical trials. In contrast to *Pf-sera5*, major immune target antigen genes of *P. falciparum*, such as apical membrane protein 1 (*ama1*), *csp* and merozoite surface antigen 1 (*msp1*), show high nucleotide diversity (θ_s_) and significant levels of positive selection (dN > dS) driven most probably by immune pressure [47] (Fig. 5). Notably, an early stage clinical trial and follow-up study in Uganda for the vaccine candidate based on the Pf-SERA5 47 kDa domain, showed that vaccination reduced clinical malaria in the vaccine cohort [48], although assessment of strain-specific response is needed.

**Fig. 6:**
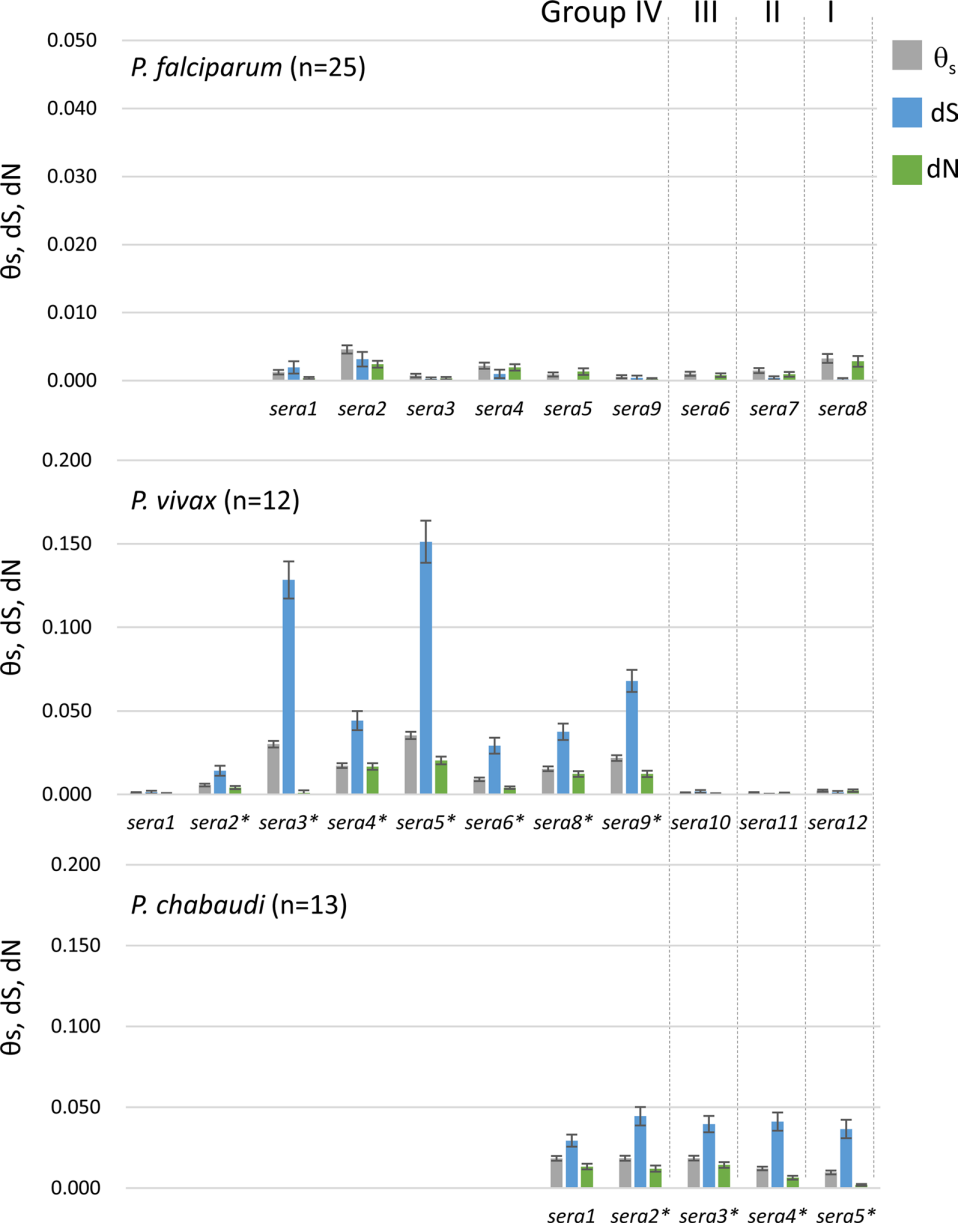
Sequence diversities (θ_s_), dS, dN in *P. falciparum*, *P. vivax*, and *P. chabaudi sera* genes. The amount of nucleotide diversity as expressed by the standardized number of polymorphic sites per site (θ_s_) were calculated using DnaSP v5.10.01 [61]. The numbers of synonymous substitutions per synonymous site (dS) and of nonsynonymous substitutions per nonsynonymous site (dN) were calculated using MEGA X [60]. As some strains of *Pv-sera7* contain stop codons in the predicted open reading frame, sequences of *Pv-sera7* were omitted from analysis. The asterisk denotes significant difference between dS and dN (*P* < 0.01)

As shown in Fig. 6, all *Pf-sera* genes exhibit low diversity/polymorphism rate, similar to *Pf-sera5*. However, *sera* genes in other *Plasmodium* species have different polymorphic features. Polymorphisms in Group IV *Plasmodium vivax (Pv)*-*sera* genes are much higher than those of Group I to III *Pv-sera* genes. The nucleotide diversities of the five *sera* genes of *Plasmodium chabaudi* (*Pc*) are similar among the gene family members. *Pc-sera* nucleotide diversity was higher than all *Pf-sera* genes and Group I to III *Pv-sera* genes; however, lower than certain Group IV *Pv-sera* genes. No significant positive selection (dN > dS) was detected for both *Pv-sera* and *Pc-sera* genes, rather it is evident that purifying selection (dS > dN) is acting on *Pv-sera* and *Pc-sera* genes marked with an asterisk in Fig. 6. The purifying selection is thought to be due to functional and structural constraints. This suggests that *sera* genes under selective pressure must be functionally active.

The major immune target antigen genes of *P. falciparum* show positive selection [47, 49–52]. In contrast, the polymorphism of *Pf-sera5* is very limited with no significant immune pressure detected. The N-terminal 47 kDa domain of Pf-SERA5 induced growth inhibitory antibodies in mice [12, 53] and humans [11, 12, 54]. Although a large amount of Pf-SERA5 is released into *P. falciparum*-infected human blood [29], the seroconversion rate of the 47 kDa domain among residents of a malaria endemic area in the Solomon Islands was much lower than that of Pf-MSP1 [14]. These data suggest that the 47 kDa fragment is less immunogenic. A recent study showed that the 47 kDa fragment bound to host vitronectin, which in turn bound to other host proteins to prevent phagocytosis of merozoites, highlighting a camouflage strategy that facilitates the parasite escape from the host immune response [55]. This phenomena was observed in the Ugandan Phase Ib trial, which reported a linear decrease in the number of vaccine responders with increasing age from 6–10 years-old, 11–15 years-old, and 16–20 years-old, with only a few vaccine responders observed in the adult cohort (21–35 years-old) [48]. In contrast, 100% seroconversion was reported in a Phase Ia trial with malaria-naive Japanese adults [14]. These observations suggest that exposure to repeated natural infections in malaria-endemic areas, as well as the ability of the 47 kDa fragment to bind to host vitronectin, dampens the immune response, thereby, causing the vaccinated individual to become ‘tolerant’. Without immune-driven selection pressure, Pf-SERA5 polymorphisms remain limited.

Differences in the number of Group IV *sera* genes are notable among primate, rodent, and avian *Plasmodium* species (Fig. 1b). In *P. berghei*, simultaneous disruption of the two Group IV *sera* genes, *Pb-sera1* and *Pb-sera2*, did not affect parasite growth [56], suggesting nonessential or auxillary roles. Similarly, disruption of *Plasmodium yoelii* (*Py*)*-sera1* and *Py-sera2*, which showed higher transcription levels in virulent compared to avirulent parasite lines, did not affect parasite survival *in vivo*, although the lethality of the parasite was attenuated [57]. In contrast, Pf-SERA5 is essential for *P. falciparum* growth [22, 27, 32–34]. These observations suggest that gene duplication in Group IV *sera* genes in primate *Plasmodium* species has occurred for functions required for host-adaptation.

## Conclusions

Of all *Plasmodium* SERA, Pf-SERA5 is currently the most studied, and was the first member to be identified before publication of the *P. falciparum* genome project. While initial studies showed Pf-SERA5 to be involved in membrane rupture and parasite egress, the cysteine protease active center of this (pseudo)protease is replaced with a serine residue, and several seminal studies have failed to show protease activity. Group IV SERA, to which Pf-SERA5 belongs, consists of several serine-type SERA resulting from repeated gene duplication events. Hence, Group IV SERA may have acquired new functions during the evolution of the malaria parasite. In *P. falciparum*, SERA5 was shown to function as a regulator of merozoite egress and was also found to interact with host proteins to evade host immunity; meanwhile, *P. yoelii* SERA2 was inferred to be associated with parasite virulence. In *P. vivax*, synonymous mutations found to accumulate in certain Group IV *sera* genes suggest their contribution to the parasite’s life-cycle. The acquisition of various functions through gene duplication would provide high parasite adaptability allowing for the expansion of host range and increased fitness. The cysteine-type SERA (Group III), Pf-SERA6 and Pb-SERA3, showed protease activity and are responsible for merozoite egress. However, the activity of Pb-SERA3 appears multifunctional, as it has been detected in the host liver and is also selectively expressed in the male gametocyte. Further, gene disruption of *Pb-sera5* (Group I SERA) demonstrated its role in sporozoite egress from oocysts. Hence, it is clear that all functions of the SERA family have not yet been elucidated and characterized, and thus, requires further investigation. In parasitology, multigene families are described primarily as being associated with immune evasion strategies by amplifying serologically different allelic types. However, the observations in the *sera* gene family suggest the possibility for other important roles of these families. The development of innovative methods would lead to an increased understanding of the SERA family.

## Supplementary information

**Additional file 1: Table S1.** Accession numbers of *Plasmodium sera* genes. Gene ID in blue can be found in PlasmoDB (https://plasmodb.org/plasmo/). Others can be found in the NCBI database (https://www.ncbi.nlm.nih.gov/). *Plasmodium sera* genes categorized as Group I, II, III, and IV are shaded in pink, yellow, green and blue, respectively.

## Data Availability

Not applicable.

## Abbreviations

*adsl*: adenylosuccinate lyase
*ama1*: apical membrane protein 1
CDPK1: calcium-dependent protein kinase 1
CSP, *csp*: circumsporozoite protein
MSP1, *msp1*: merozoite surface antigen 1
*Pb*: *Plasmodium berghei*
*Pc*: *Plasmodium chabaudi*
*Pf*: *Plasmodium falciparum*
*Pg*: *Plasmodium gallinaceum*
*Pr*: *Plasmodium relictum*
*Pv*: *Plasmodium vivax*
PV: parasitophorous vacuole
PVM: parasitophorous vacuole membrane
*Py*: *Plasmodium yoelii*
*serca*: P-type Ca^2+^-ATPase
SERA, *sera*: serine repeat antigen
SUB1: subtilisin-like serine protease 1
